# Rise of the (possible) resistance: a review of susceptibility patterns for nontyphoidal *Salmonella enterica* in Nebraska

**DOI:** 10.1017/ash.2023.464

**Published:** 2023-10-23

**Authors:** Shawnalyn W. Sunagawa, Catherine Christopherson, Scott J. Bergman, Molly M. Miller, Mackenzie R. Keintz, Taylor A. Wahlig, Paul Fey, Trevor C. Van Schooneveld

**Affiliations:** 1 Department of Pharmaceutical and Nutrition Care, Nebraska Medicine, Omaha, NE, USA; 2 College of Pharmacy, University of Nebraska Medicine Center, Omaha, NE, USA; 3 Division of Infectious Diseases, University of Nebraska Medical Center, Omaha, NE, USA; 4 Department of Pathology and Microbiology, University of Nebraska Medical Center, Omaha, NE, USA

## Abstract

Our institution sought to evaluate our antimicrobial stewardship empiric treatment recommendations for *Salmonella*. Results from 36 isolates demonstrated reduced susceptibilities to fluoroquinolones with 1 isolate susceptible only to ceftriaxone. Analysis supports the current recommendation of empiric ceftriaxone therapy for severe infection and updated recommendation for sulfamethoxazole-trimethoprim in non-severe infections.

## Background

Nontyphoidal *Salmonella enterica* is a common foodborne pathogen that accounts for approximately 1.35 million illnesses and roughly 400 deaths annually in the United States.^1^ Illness is usually self-limited and presents as gastroenteritis, although invasive infections occasionally occur and are associated with increased morbidity and mortality, especially in immunocompromised patients.^2^ In 2021, a Centers for Disease Control and Prevention review of *Salmonella* susceptibilities demonstrated a 40% increase in the annual incidence of infections with resistance to either ampicillin, ceftriaxone, or ciprofloxacin, with over half of the increase attributable to ciprofloxacin non-susceptible isolates.^3^ Furthermore, the Clinical and Laboratory Standards Institute lowered their minimum inhibitory concentration breakpoints for *Salmonella spp.* in response to these resistance patterns.^4^

Nebraska Medicine’s Clinical Microbiology laboratory has utilized the FilmArray Gastrointestinal Panel (GIP) to identify pathogens such as *Salmonella enterica* in the stool since 2014. This test was implemented in conjunction with treatment guidance from the Antimicrobial Stewardship Program (ASP).^5^ The GIP detects 22 pathogens within 1–2 hours, but antibiotic susceptibility data are not routinely available using this method since cultures are not routinely obtained. Therefore, we assessed the susceptibility of *Salmonella* spp. isolates detected by culture at our regional reference and local laboratory to determine the appropriateness of ASP’s current recommendations for treating severe/invasive disease/bacteremia with ceftriaxone and non-severe infections with fluoroquinolones or trimethoprim-sulfamethoxazole.

## Methods

A retrospective cohort of all *Salmonella* spp. isolates from both our regional (Nebraska and Western Iowa) referral and local laboratory from 1/1/2020 to 11/1/2022 were reviewed. Patient demographics, *Salmonella* spp. serogroup, culture site, and antibiotic susceptibilities by disk diffusion (stool isolates) or Microscan (non-stool isolates) were assessed. Descriptive statistics were utilized to summarize the data. This study was deemed a quality improvement project and exempted from review by our institutional review board.

## Results

**Figure 1.**
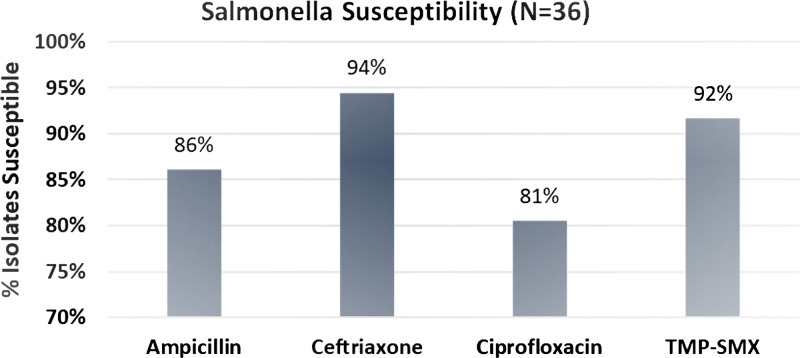
*Salmonella* isolate susceptibilities.

There were 887 GIP tests positive for *Salmonella* spp., with a majority (*n* = 756, 85.2%) sent to our referral laboratory from outside facilities. Altogether, there were a total of 36 *Salmonella* spp. isolates from 36 unique patients with susceptibility data available for review, of which 17 (47.2%) had been sent to our laboratory from outside facilities. Most (*n* = 25, 69.4%) were from female patients, and the overall cohort had an average age of 54.5 years (Table 1). A majority of isolates with susceptibilities were from blood cultures (*n* = 19, 52.8%), with the others being from urine (*n* = 13, 36.1%), stool (*n* = 3, 8.3%), and wound (*n* = 1, 2.8%). Of these 887 GIP stool samples, only 3 had susceptibilities requested and reported. Serogroups were identified in 28 isolates (77.8%), with Group C most common (*n* = 12, 33.3%). Additional cohort characteristics are listed in Table 1. Results indicated that 86.1% of isolates were susceptible to ampicillin, 94.4% to ceftriaxone, 80.6% to ciprofloxacin, and 91.7% to trimethoprim-sulfamethoxazole (Figure 1). No isolate was resistant to all reported antibiotics; however, 1 isolate (from urine culture) was only susceptible to ceftriaxone.

**Table 1.** Cohort characteristics and isolate susceptibilities

Cohort characteristics (*N* = 36)	*N* (%)
Female	25 (69.4%)
Age (years), average ± SD	54.5 ± 21
Collection location	
Nebraska Medical Center	19 (52.8%)
Referral facility	17 (47.2%)
Culture site	
Blood	19 (52.8%)
Urine	13 (36.1%)
Stool	3 (8.3%)
Wound	1 (2.8%)
Microbiology Lab Reported Salmonella Serotype	
Group B	7 (19.4%)
Group C	12 (33.3%)
Group C/D	3 (8.3%)
Group D	6 (16.7%)
No serotype	8 (22.2%)

Note. SD, standard deviation.

## Discussion

Our analysis supports the finding of increasing resistance in *Salmonella* spp. isolates, particularly fluoroquinolones.^3,6^ It is a concerning trend that approximately 20% of our available isolates were not susceptible to ciprofloxacin, especially since the incidence of gastroenteritis has not significantly changed in the past decade.^7^ Additionally, projected 2022 data from the National Antimicrobial Resistance Monitoring System showed 84% of *Salmonella* spp. isolates were non-susceptible to ciprofloxacin,^6^ which would argue against the utilization of a fluoroquinolone as an empiric treatment agent. However, this study also validated previously published local data showing low prevalence of *Salmonella* spp. with resistance to both ceftriaxone and fluoroquinolone in Nebraska (0% in 2010–2013 to 2.8% in 2015).^8^ This analysis provides insight into overall changes in our local susceptibility patterns, in relation to national susceptibility trends, which is relevant since a vast majority of *Salmonella* spp. infections are now identified through molecular testing and do not have susceptibilities performed. As rapid molecular diagnostics become the main method to identify organisms, this study highlights the importance of continuing to review local susceptibility patterns since they may vastly differ from national trends and can impact local empiric treatment recommendations. With this information, we were able to validate our ASP guidance for the recommendation of empiric ceftriaxone in severe/invasive disease/bacteremia. However, our local recommendation for an oral treatment in non-severe disease was updated to sulfamethoxazole-trimethoprim as the preferred agent in patients with GIP positive for *Salmonella* spp. Finally, while empiric combination therapy with ceftriaxone and fluoroquinolone may not be necessary for severe/invasive infections based on local resistance data, it remains an option for those not responding to ceftriaxone alone.

This was a small retrospective cohort review; however, it highlights the real-world change in practice on the diagnosis and treatment of *Salmonella* spp. infections since a majority of infections are now identified through molecular testing. While susceptibilities are routinely performed for all isolates from blood, urine, and wound cultures, we acknowledge the potential for selection bias on the stool culture isolates since susceptibilities are only performed when requested. This may lead to susceptibilities being requested in patients who are not improving on empiric therapy and thus, potentially having more resistant isolates. However, by reviewing overall susceptibility patterns, we were able to tailor our ASP recommendations based on local resistance. Additionally, we acknowledge that we did not characterize the mechanisms of resistance for the *Salmonella* spp. isolates. Nationally, beta-lactam resistance has been shown to be primarily mediated through *bla*_CMY-2_^8^; however, Murphy et al. previously sequenced isolates in Nebraska and characterized that beta-lactam resistance was mediated through *bla*_CTX-M_.^9^ Fluoroquinolone resistance was mediated through both the presence of PMQR and QRDR genes.^9,10^ While the main causative mechanisms of resistance for *Salmonella* spp. may change over time or vary by region, our data supports increasing resistance, which is not detected by molecular testing and can cause challenges for the treatment of patients with severe/invasive infection.

Based on our local, real-world *Salmonella* spp. susceptibility data, our ASP recommendation for empiric ceftriaxone in severe/invasive diseases/bacteremia is still appropriate since 94% of isolates were susceptible to ceftriaxone. However, trimethoprim-sulfamethoxazole is the preferred empiric oral therapy for non-severe disease due to locally observed increasing resistance to fluoroquinolones that confirms previously published data.^3,6^ Further study of additional isolates, drug susceptibility patterns, and mechanisms of resistance are warranted to better assess the ideal therapy in our region where most testing for *Salmonella* spp. is currently being performed by molecular methods. This report highlights and further emphasizes the concerning trend and spread of isolates with non-susceptibility to first-line antimicrobial therapies, which is an ongoing public health concern.
